# Guiding the Way: Organizational Transparency as a Statistical Mediator in the Association Between Quantum Leadership and Organizational Readiness for Change Among Nurses

**DOI:** 10.1155/jonm/9937065

**Published:** 2026-08-12

**Authors:** Amal Diab Ghanem Atalla, Ayman Mohamed El-Ashry, Mohamed Saad Saleh Ali, Duaa Amr Hafez, Makiah Mohammed Shebaili, Ebaa Marwan Felemban, Faygah Shibily, Wafaa Hassan Mostafa

**Affiliations:** ^1^ Department of Nursing Administration, Faculty of Nursing, Alexandria University, Alexandria, Egypt, alexu.edu.eg; ^2^ Department of Psychiatric and Mental Health Nursing, Faculty of Nursing, Alexandria University, Alexandria, Egypt, alexu.edu.eg; ^3^ Department of Nursing, College of Applied Medical Sciences, Jouf University, Al Qurayyat, Saudi Arabia, ju.edu.sa; ^4^ Public Health Nursing Department, College of Nursing, Northern Border University, Arar, Saudi Arabia, nbu.edu.sa; ^5^ Public Health Nursing Department, Faculty of Nursing, King Abdulaziz University, Jeddah, Saudi Arabia, kau.edu.sa; ^6^ Critical Care Nursing Department, Faculty of Nursing, King Abdulaziz University, Jeddah, Saudi Arabia, kau.edu.sa; ^7^ Department of Nursing Administration, Faculty of Nursing, Damanhour University, El-Behira, Egypt, damanhour.edu.eg

**Keywords:** mediator, nurses, organizational transparency, quantum leadership, readiness for change

## Abstract

**Aim:**

To assess levels of quantum leadership, organizational transparency, and organizational readiness for change among staff nurses and to examine whether organizational transparency statistically mediates the association between quantum leadership and organizational readiness for change.

**Design:**

A cross‐sectional descriptive study was reported in accordance with STROBE guidelines.

**Methods:**

A census sampling approach was used to recruit 350 staff nurses from Shoubrakhit General Hospital. Data were collected using the Quantum Leadership Survey, the Organizational Transparency Instrument, and the Organizational Readiness for Implementing Change Scale.

**Results:**

Most nurses reported high levels of quantum leadership (77.1%), organizational transparency (66.3%), and readiness for change (85.4%). Correlation analysis showed positive associations among the three main variables. The statistical mediation model indicated that quantum leadership was directly associated with organizational readiness for change and showed a small positive indirect association through organizational transparency. The model demonstrated acceptable overall fit.

**Conclusion:**

Quantum leadership was positively associated with organizational readiness for change, with organizational transparency showing a small statistical mediating role. Given the cross‐sectional, self‐report, single‐hospital design, the findings should be interpreted cautiously as associations rather than evidence of causal processes.

**Implications for Nursing Management:**

Nurse managers and nursing leaders should strengthen transparent communication channels, involve nurses meaningfully in committees and change‐related discussions, support accountable decision‐making, and assess staff readiness before major organizational initiatives.

## 1. Introduction

Healthcare delivery has become increasingly complex because of changing nursing roles and growing patient needs. These challenges underscore the relevance of skilled nursing leadership to the management of dynamic healthcare systems and to the maintenance of supportive work environments [1]. In the healthcare industry, the modern leadership approach called “quantum leadership” is applied. It is referred to as “a framework for managing an organization to transform it into an exceptional organization regarding performance, productivity, and profitability, and as a combination of abilities that transform leaders and organizations to promote innovation” [2].

In nursing and organizational research, transformational leadership and complexity‐informed leadership remain among the most extensively studied and empirically supported approaches; however, emerging perspectives intended to address uncertainty and complexity in modern healthcare systems are receiving greater attention. One such perspective is quantum leadership. Rather than replacing established leadership theories, quantum leadership emphasizes flexibility, interconnectedness, teamwork, and adaptation to changing circumstances. Although the framework remains less established and has received limited empirical investigation in nursing, it may provide an additional perspective for examining how leaders respond to uncertainty, encourage creativity, and support organizational transformation. It is therefore relevant to investigate whether quantum leadership is associated with organizational transparency and readiness for change without assuming that it is superior to established approaches [3].

Quantum leadership is commonly described in terms of two dimensions. Quantum leadership skills include the leader’s capacity to involve and guide staff in decision‐making, support innovative ideas and planning, respond to fear with appreciation and compassion, and approach complex problems creatively [4]. Quantum leadership characteristics include self‐management, attention to the safety of others, exploration of novel organizational solutions, revision of strategic plans, recognition of employees′ professional development, support for a sense of organizational community, and encouragement of individual potential for innovation [5].

Quantum leadership has been proposed as a set of practices that may be associated with staff perceptions of security, mutual trust, communication, learning, autonomy, and alignment with organizational objectives [6, 7]. Its emphasis on adaptability and collaborative problem‐solving may also be relevant to organizational readiness for change. However, these propositions require empirical examination, particularly in nursing settings. Quantum leaders may support readiness by communicating a clear vision, encouraging staff participation, and considering long‐term organizational needs [8].

Organizational readiness for change refers to the behavioral and psychological preparedness of organizational members to implement change [9]. Its two main dimensions are change efficacy, or members′ shared confidence in their collective ability to implement change, and change commitment, or their shared resolve to pursue implementation [10, 11]. Higher readiness has been associated with greater effort, persistence, cooperation, and participation in implementing and evaluating new interventions, although these relationships may vary across contexts [12, 13]. Organizational transparency may also be relevant during periods of change because access to clear information and opportunities for participation may be associated with lower uncertainty and greater understanding of proposed initiatives. Previous literature suggests that transparency may be related to lower perceived uncertainty and job insecurity during organizational change [14, 15].

Organizational transparency refers to the open exchange of information concerning organizational knowledge, decisions, and procedures [16]. Its dimensions include overall perceived transparency, substantial information, participation, accountability, and secrecy as a reverse indicator of openness [17]. Overall perceived transparency reflects how individuals evaluate openness in organizational communication. Information substantiality refers to the timely provision of relevant, understandable, complete, accurate, dependable, and verifiable information. Participation concerns opportunities to identify and communicate stakeholder needs, while accountability involves acknowledging shortcomings and identifying areas for improvement. Secrecy refers to the deliberate concealment of relevant information or conduct [18]. Organizational transparency has been linked to perceptions of accountability and trust and may be associated with stakeholder relationships, organizational performance, and staff morale [19].

### 1.1. Significance of the Study

This study addresses a relevant issue within the Egyptian healthcare context, where organizations may face limited resources, workforce shortages, and changing patient and disease profiles. Effective leadership and organizational capacity for change may be relevant to managing this complexity and supporting the delivery of patient care. Quantum leadership may offer an additional framework for examining empowering, flexible, and collaborative leadership attributes in healthcare. By examining its association with organizational readiness for change and the statistical mediating role of transparency, this study provides context‐specific evidence that may inform nursing leaders and healthcare managers.

Organizational transparency is another relevant consideration. Trust, communication, and employee involvement have been associated with staff responses to organizational change; therefore, examining transparency may be particularly useful in settings characterized by hierarchical organizational structures. The findings concerning statistical mediation may inform discussions about communication, participation, and accountability practices that could support organizational readiness. The focus on nurses is also important because nurses are closely involved in implementing policies, procedures, technologies, and service changes. Examining these relationships among Egyptian nurses may provide practical information for nursing management while recognizing that the study does not establish causal effects.

A comprehensive examination of the association between quantum leadership and organizational readiness for change, with organizational transparency specified as a statistical mediator, has been limited in the Egyptian nursing context. The present study addresses this knowledge gap.

### 1.2. Conceptual Framework of the Study

**FIGURE 1:**
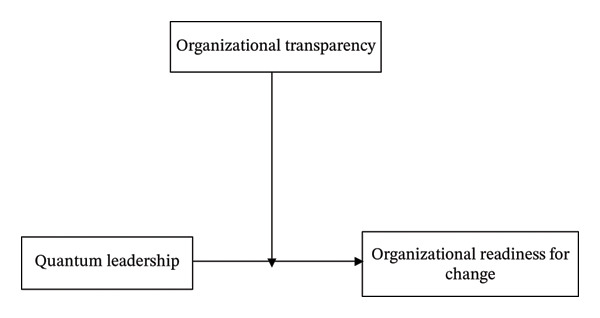
Proposed statistical mediation framework for the associations among quantum leadership, organizational transparency, and organizational readiness for change.

This study was guided by the statistical mediation framework illustrated in Figure 1. Organizational transparency was specified as a statistical mediator in the association between quantum leadership and organizational readiness for change. Quantum leadership was specified as the predictor variable, organizational readiness for change as the outcome variable, and organizational transparency as the intervening statistical variable. The model estimated the direct association between quantum leadership and readiness for change and the indirect association through organizational transparency. These paths represent statistical associations and do not establish temporal order or causality.

### 1.3. Theoretical Framework of the Study

Weiner’s Organizational Readiness for Change theory, which conceptualizes readiness in terms of shared change commitment and change efficacy among organizational members, informed this study [9]. Within this perspective, leadership practices and organizational conditions may be related to how employees perceive and prepare for change. Quantum leadership, characterized by creativity, flexibility, and collaborative decision‐making, was therefore examined as a leadership construct that may be associated with readiness for change. Perspectives on transparency and information processing were also considered because access to timely, accurate, and relevant information may be associated with lower uncertainty, greater trust, and stronger understanding during change processes. In the tested model, organizational transparency was specified as a potential statistical mediator of the association between quantum leadership and organizational readiness for change. This specification represents an indirect statistical association rather than a confirmed causal mechanism, particularly because all variables were measured at one time point [18].

### 1.4. Aim of the Study

• To examine the associations among quantum leadership, organizational transparency, and organizational readiness for change and to test whether organizational transparency statistically mediates the association between quantum leadership and organizational readiness for change.

### 1.5. The Research Question

• What are the associations among quantum leadership, organizational transparency, and organizational readiness for change, and does organizational transparency show statistical mediation in the association between quantum leadership and organizational readiness for change?

## 2. Methods

### 2.1. Study Design

A cross‐sectional descriptive design was chosen in compliance with STROBE principles.

### 2.2. Setting

This study was conducted at the 200‐bed Shoubrakhit General Hospital in collaboration with the Ministry of Health and Population (MOHP). This hospital is thought to be among the best medical facilities in the El‐Beheira Governorate. All intensive care units (ICUs) and medical‐surgical inpatient care units (*N* = 13) were included in the study. There are two types of inpatient care units: ICUs (*n* = 4), which include general, neonate, pediatric, and medical units (*n* = 5), including obstetric, pediatric, fever, and general medical (A and B); emergency care units; and surgical units (*n* = 4), including dialysis, operation, and general surgical (A and B).

### 2.3. Sampling

The total eligible nursing population available across the pilot and main‐study phases was 385 nurses. A separate group of 35 eligible nurses participated in the pilot study and was excluded from the main study. The remaining 350 eligible nurses constituted the census population for the main study. All 350 eligible nurses were invited to participate in the main study. Eligibility criteria included the following: (1) working in one of the selected clinical units; (2) having at least 1 year of experience in the current unit; and (3) providing direct patient care. All invited nurses agreed to participate, completed the questionnaires, and were included in the final analysis. Therefore, the final analyzed sample was 350 nurses, and the response rate was 100% (350/350).

### 2.4. Ethical Considerations

On March 21, 2024, the study was formally approved by the Faculty of Nursing Research Ethics Committee. The Faculty of Nursing Research Ethics Committee at Damanhur University in Egypt formally approved this study with reference code DAMANHOUR/98/c/2024. Additionally, nurses who decided to take part in the study provided written informed consent before they participated in the study. Participation was entirely voluntary, and participants were free to decline or withdraw at any point without any consequences. Each questionnaire had a code number attached to it to guarantee confidentiality and identity protection. The nurses received assurances that the study would be the only use of the data. It has been shown that nurses can withdraw from research.

### 2.5. Study Tools

Among other things, the researchers inquired about the study participants′ age, gender, years of service, years spent in the work unit, education, and nursing experience.

### 2.6. Quantum Leadership Survey

Oidem et al. [1] created the quantum leadership survey to learn more about making decisions and addressing problems, as well as participants′ opinions of the quantum leadership style. It consists of 20 items split between two dimensions: Quantum Leadership Characteristics and Quantum Leadership Skills (10 items for each dimension). On a 5‐point Likert rating scale that went from 1 (*strongly disagree*) to 5 (*strongly agree*), the participants′ responses to the questionnaire were evaluated. Between 20 and 100 is the range of the overall mean score. Cronbach’s alpha for the current study was 0.90. The researchers used an exploratory factor analysis to evaluate the tool’s validity. Before and after varimax rotation, the loading scores varied between 0.512 and 0.966 and 0.544 and 0.951, respectively. This was greater than 0.35 and accounted for 82.151% of the total variation. Kaiser–Meyer–Olkin determined that the data were suitable for factor analysis with a sampling adequacy of 0.923. Moreover, the statistical significance (*p* < 0.001) found by Bartlett’s test of sphericity validated the factor capacity of the correlation matrix. Consequently, the parts of the scale were retained. The possible overall raw score ranged from 20 to 100, with higher scores indicating greater perceived quantum leadership.

### 2.7. Organizational Transparency Scale

The organizational transparency scale was established by Al‐Muqati et al. [17]. There are five subscales in the transparency area, with a total of 27 items. The organization’s overall perceived openness is measured by four questions, involvement by six, substantial information by seven, accountability by five, and concealment by five reverse‐scored items [17].

The replies were scored using a 5‐point Likert scale, which goes from (*strongly disagree*) 1 to (*strongly agree*) 5. The overall score ranges from 27 to 135 points. Cronbach’s alpha for this study was 0.93. An exploratory factor analysis was used by the researchers to assess the tool’s validity. The loading scores ranged from 0.662 to 0.912 before varimax rotation and from 0.598 to 0.982 after varimax rotation. This explained over 0.45, or 79.45%, of the total variation. The data were suitable for factor analysis, as shown by the Kaiser–Meyer–Olkin sampling adequacy of 0.923. Furthermore, the factor capacity of the correlation matrix was confirmed by Bartlett’s test of sphericity, which achieved statistical significance (*p* < 0.001). Consequently, the elements on the scale were retained. The possible overall raw score ranged from 27 to 135. The secrecy items were reverse‐coded before the total score was calculated; therefore, higher total scores indicated greater perceived organizational transparency.

### 2.8. The Organizational Readiness for Implementing Change Scale (ORIC)

Shea et al. [20] created the ORIC to gauge the perceived level of organizational preparation for change implementation, drawing on Weiner’s organizational theory [9]. The 12‐item Likert scale assessment, ORIC, is a dependable multilevel construct that highlights commitment and change efficacy. Organizational members′ common resolve to effect change is demonstrated by change commitment (5 statements), while their mutual belief in their capacities to do so is demonstrated by change efficacy (7 items). These 12 items are rated from “*Disagree*” to “*Agree*” on a 5‐point Likert scale. The clinician’s principal occupation and workplace were the subject of two additional questions.

The overall score ranges from 12 to 60. The current study’s Cronbach’s alpha was 0.82. The researchers used an exploratory factor analysis to assess the tool’s validity. The loading score varied between 0.541 and 0.91 before the varimax rotation and between 0.501 and 0.902 after the rotation. The fact that it explained 80.151% of the total variation made it more significant than 0.35. The Kaiser–Meyer–Olkin measure indicated that the data may be used for factor analysis, with a sample adequacy of 0.903. Furthermore, the capacity of the correlation matrix was confirmed by Bartlett’s test of sphericity, which achieved statistical significance (*p* < 0.001). Consequently, the elements on the scale were retained. The possible overall raw score ranged from 12 to 60, with higher scores indicating greater perceived organizational readiness for change.

### 2.9. Scoring System

For each instrument and its dimensions, item scores were summed to obtain a raw total score. Before calculating the Organizational Transparency Scale scores, the five negatively worded secrecy items were reverse‐coded so that higher scores consistently indicated greater organizational transparency. To facilitate comparison across instruments with different numbers of items, raw scores were converted into percentage‐of‐possible scores using the following formula:

(1) Percentage score=observed raw score−minimum possible scoremaximum possible score−minimum possible score×100.

The developers of the instruments did not provide validated diagnostic or clinical cutoff points for classifying respondents as having low, moderate, or high levels. Therefore, for descriptive purposes, the researchers divided the possible percentage‐score range into three equal intervals: low, 0 to < 33.3%; moderate, 33.3 to < 66.6%; and high, ≥ 66.6%. These categories were established a priori and were based on the possible scale range rather than the distribution of scores in the present sample. They should be interpreted as descriptive categories and not as validated clinical or diagnostic thresholds. Continuous total and mean percentage scores were retained as the primary measures in the statistical analysis.

### 2.10. Pilot Study

A pilot study was conducted with 35 nurses, representing 10% of the planned main‐study sample. The pilot participants met the same eligibility criteria as the main‐study participants but were not included in the final analysis. The pilot study was undertaken to evaluate the clarity, relevance, comprehensiveness, and feasibility of the questionnaires and the data‐collection procedures. After completing the questionnaires, the pilot participants were asked to provide feedback on the clarity of the instructions and individual items, the appropriateness of the wording, the relevance of the questions to the study constructs, the completeness of the content, and any difficulty experienced while responding. The research team also reviewed the completed questionnaires for unanswered items, inconsistent responses, repeated misunderstanding of items, and practical difficulties related to questionnaire administration.

Feasibility was assessed by examining the time required to complete the questionnaires, participants’ ability to understand and answer the items without substantial assistance, and the practicality of distributing and collecting the questionnaires within the clinical units. The average completion time was approximately 15–20 min.

Based on feedback from the pilot participants and the research team’s review, the questionnaires were considered clear, relevant, sufficiently comprehensive, and feasible for use in the main study. No items were added, deleted, or reworded, and no changes were made to the scoring procedures or data‐collection process. The 35 pilot participants were excluded from the main‐study sample.

### 2.11. Common Method Bias Assessment

Because all study variables were measured using self‐report questionnaires administered at a single time point, the possibility of common method variance was considered. Harman’s single‐factor test was conducted as a preliminary diagnostic assessment. The first unrotated factor accounted for less than 50% of the total variance, indicating that no single dominant factor was evident. However, Harman’s single‐factor test cannot rule out common method variance, and the possibility of common method bias cannot be fully excluded.

Procedural measures were used to reduce potential response bias, including assuring participants of confidentiality, emphasizing voluntary participation, providing standardized instructions, and using previously validated measurement instruments. Nevertheless, the findings should be interpreted cautiously because the predictor, mediator, and outcome variables were collected from the same respondents using self‐report measures at one point.

### 2.12. Data Collection

Data were collected over 3 months, from April to June 2024. Following completion of the pilot study, the researchers coordinated with the nurse managers of the selected clinical units to identify nurses who met the eligibility criteria. Eligible nurses were approached individually at suitable times that did not interfere with patient care. The researchers explained the study purpose, procedures, voluntary nature of participation, confidentiality safeguards, and the nurses’ right to decline participation or withdraw at any time without penalty.

After providing written informed consent, each participant received a coded paper questionnaire and standardized instructions for completion. The explanation and instructions required approximately 2 min. No names or directly identifying information were recorded on the questionnaires. Participants completed the questionnaires individually in a suitable area within their clinical units. A member of the research team remained available to answer procedural questions without interpreting the questionnaire items or influencing participants’ responses.

Questionnaire distribution and return were monitored within each clinical unit by recording the number of eligible nurses approached, questionnaires distributed, and completed questionnaires returned. Eligible nurses who were unavailable during the initial visit because of shift schedules, leave, workload, or clinical responsibilities were approached during subsequent visits conducted at different times and on different working days. These follow‐up visits were intended to provide nurses working across different shifts with an equal opportunity to participate. Participation remained voluntary, and no pressure was placed on nurses who declined or had not yet responded.

The questionnaires required approximately 15–20 min to complete. Participants returned the completed questionnaires directly to a member of the research team. Each questionnaire was reviewed upon return to identify unintentionally omitted items. When an item was left unanswered, the participant was informed and asked whether they wished to complete it; participants were free to leave any item unanswered.

All 350 eligible nurses invited to participate in the main study returned complete questionnaires, and all questionnaires were included in the final analysis. Therefore, the final analyzed sample comprised 350 nurses, corresponding to a response rate of 100% (350/350).

After returning the complete questionnaire, participants were offered bottled water as a modest expression of appreciation for their time. The refreshments were provided only after questionnaire completion, were not announced during recruitment, were not conditional on answering all questionnaire items, and were not presented as payment or compensation. Their provision was approved as part of the study protocol reviewed by the Research Ethics Committee. Given their minimal value and the fact that they were provided after participation, they were not considered likely to exert undue influence on nurses’ decisions to participate.

### 2.13. Data Analysis

The data were transferred onto the computer and analyzed using IBM SPSS software Version 23.0. One method was to employ an ANOVA test when comparing more than two categories. Two quantitative data categories that were consistently distributed were compared using Student′s *t*‐test. The Pearson coefficient was used to correlate quantitative data that were spread out over time. Statistical significance was set at *p* ≤ 0.05. The internal consistency of the scale was assessed using Cronbach’s α coefficient.

## 3. Results

Table 1 shows that 87.1% of nurses are female and 68.0% are between the ages of 30 and 40. Furthermore, 76.3% of nurses are married. Furthermore, 18.9%, 33.7%, and 34.6% are employed in surgical, critical care, and medical units. 42.0% have a Bachelor of Nursing. 38.9% have 10 to 15 years of experience in nursing. 39.1% have from 10 to 15 years of experience in units. 88.0%, 100%, and 88.0% of nurses have no previous training programs about quantum leadership, organizational change readiness, and organizational transparency, respectively. 54.3%, 88.0%, and 76.6% of nurses need training programs about quantum leadership, organizational readiness for implementing change, and organizational transparency, respectively.

**TABLE 1:** Distribution of the studied nurses according to demographic data (*n* = 350).

Demographic characteristics	No.	%
Sex		
Male	45	12.9
Female	305	87.1
Age (years)		
30–40	238	68.0
40–50	112	32.0
Marital status		
Married	267	76.3
Divorced	4	1.1
Widowed	79	22.6
Unit		
Medical	121	34.6
Surgical	66	18.9
Critical care	118	33.7
Other	45	12.9
Education level		
Diplomat of nursing	22	6.3
Technical institute	143	40.9
Bachelor of nursing	147	42.0
Fellowship	38	10.9
Experience in nursing		
< 5	25	7.1
5–10	106	30.3
10–15	136	38.9
15–20	73	20.9
More than 20	10	2.9
Experience in the unit		
< 5	27	7.7
5–10	105	30.0
10–15	137	39.1
15–20	73	20.9
More than 20	8	2.3
Previous training in quantum leadership		
No	308	88.0
Yes	42	12.0
Need training in quantum leadership		
No	160	45.7
Yes	190	54.3
Previous training of organizational readiness for implementing change		
No	350	100.0
Yes	0	0.0
Need training in organizational readiness for implementing change		
No	42	12.0
Yes	308	88.0
Previous training on organizational transparency		
No	308	88.0
Yes	42	12.0
Need training on organizational transparency		
No	82	23.4
Yes	268	76.6

Table 2 shows that most nurses reported high perceived levels of quantum leadership (77.1%), organizational transparency (66.3%), and organizational readiness for implementing change (85.4%). Organizational readiness for implementing change had the highest mean percentage score (Mean ± SD = 73.59 ± 7.92).

**TABLE 2:** Distribution of the studied nurses according to their levels and mean percent score (*n* = 350).

	Low (< 33.3%)		Moderate(33.3–<66.6%)		High (≥ 66.6%)		Total score	Mean percent score	Mean score
	No.	%	No.	%	No.	%	Mean ± SD	Mean ± SD	Mean ± SD
Quantum leadership instrument									
(A) Quantum leadership skills dimension	0	0.0	50	14.3	300	85.7	38.51 ± 1.68	71.26 ± 4.21	3.85 ± 0.17
(B) Quantum leadership characteristics	0	0.0	113	32.3	237	67.7	37.12 ± 1.96	67.79 ± 4.91	3.71 ± 0.20
Overall quantum leadership	0	0.0	80	22.9	270	77.1	75.62 ± 3.31	69.53 ± 4.14	3.78 ± 0.17
Organizational transparency (OT) instrument									
(A) Overall transparency	2	0.6	122	34.9	226	64.6	14.55 ± 2.17	65.95 ± 13.57	3.64 ± 0.54
(B) Participation	0	0.0	139	39.7	211	60.3	21.72 ± 2.03	65.51 ± 8.44	3.62 ± 0.34
(C) Substantial information	0	0.0	98	28.0	252	72.0	25.65 ± 2.26	66.60 ± 8.09	3.66 ± 0.32
(D) Accountability	0	0.0	266	76.0	84	24.0	18.04 ± 1.23	65.21 ± 6.14	3.61 ± 0.25
(E) Secrecy	0	0.0	117	33.4	233	66.6	18.68 ± 1.42	68.41 ± 7.09	3.74 ± 0.28
Overall (OT)	0	0.0	118	33.7	232	66.3	98.65 ± 7.57	66.34 ± 7.22	3.65 ± 0.29
Organizational readiness for implementing change (ORIC)									
(A) Change commitment	0	0.0	39	11.1	311	88.9	20.0 ± 2.05	75.0 ± 10.23	4.0 ± 0.41
(B) Change efficacy	0	0.0	46	13.1	304	86.9	27.21 ± 2.32	72.18 ± 8.30	3.89 ± 0.33
Overall ORIC	0	0.0	51	14.6	299	85.4	47.21 ± 3.73	73.59 ± 7.92	3.94 ± 0.32

*Note:* Level classifications were based on percentage‐of‐possible scores calculated as follows: [(observed score − minimum possible score)/(maximum possible score − minimum possible score)] × 100. Scores were categorized descriptively as low (0 to < 33.3%), moderate (33.3 to < 66.6%), or high (≥ 66.6%). These categories represent equal divisions of the possible score range and are not validated clinical cutoff points.

Table 3 presents the correlation matrix among the study variables. The correlations among the main overall variables were positive and statistically significant. Overall quantum leadership was positively correlated with organizational transparency (*r* = 0.123, *p* = 0.021) and organizational readiness for change (*r* = 0.206, *p* < 0.001). Additionally, a small but statistically significant positive correlation (*r* = 0.205, *p* < 0.001) was observed between organizational readiness for change and organizational transparency.

**TABLE 3:** Correlation between the studied variables (*n* = 350).

		Quantum leadership skills dimension	Quantum leadership characteristics	Overall quantum leadership	Overall transparency	Participation	Substantial information	Accountability	Secrecy	Overall (OT)	Change commitment	Change efficacy
Quantum leadership skills dimension	*r*											
	*p*											

Quantum leadership characteristics	*r*	0.649^∗^										
	*p*	< 0.001^∗^										

Overall quantum leadership	*r*	0.893^∗^	0.922^∗^									
	*p*	< 0.001^∗^	< 0.001^∗^									

Overall transparency	*r*	0.115^∗^	0.158^∗^	0.152^∗^								
	*p*	0.032^∗^	0.003^∗^	0.004^∗^								

Participation	*r*	0.088	0.067	0.084	0.688^∗^							
	*p*	0.099	0.214	0.115	< 0.001^∗^							

Substantial information	*r*	0.087	0.123^∗^	0.118^∗^	0.741^∗^	0.746^∗^						
	*p*	0.103	0.021^∗^	0.028^∗^	< 0.001^∗^	< 0.001^∗^						

Accountability	*r*	0.062	0.024	0.046	0.580^∗^	0.608^∗^	0.516^∗^					
	*p*	0.250	0.654	0.396	< 0.001^∗^	< 0.001^∗^	< 0.001^∗^					

Secrecy	*r*	0.105^∗^	0.043	0.079	0.592^∗^	0.417^∗^	0.399^∗^	0.599^∗^				
	*p*	0.049^∗^	0.420	0.141	< 0.001^∗^	< 0.001^∗^	< 0.001^∗^	< 0.001^∗^				

Overall (OT)	*r*	0.112^∗^	0.112^∗^	0.123^∗^	0.897^∗^	0.865^∗^	0.870^∗^	0.758^∗^	0.685^∗^			
	*p*	0.036^∗^	0.036^∗^	0.021^∗^	< 0.001^∗^	< 0.001^∗^	< 0.001^∗^	< 0.001^∗^	< 0.001^∗^			

Change commitment	*r*	0.101	0.124^∗^	0.125^∗^	0.128^∗^	0.145^∗^	0.119^∗^	0.248^∗^	0.014	0.154^∗^		
	*p*	0.060	0.020^∗^	0.020^∗^	0.017^∗^	0.006^∗^	0.026^∗^	< 0.001^∗^	0.797	0.004^∗^		

Change efficacy	*r*	0.173^∗^	0.223^∗^	0.220^∗^	0.196^∗^	0.153^∗^	0.217^∗^	0.086	0.093	0.193^∗^	0.454^∗^	
	*p*	0.001^∗^	< 0.001^∗^	< 0.001^∗^	< 0.001^∗^	0.004^∗^	< 0.001^∗^	0.108	0.081	< 0.001^∗^	< 0.001^∗^	

Overall ORIC	*r*	0.163^∗^	0.207^∗^	0.206^∗^	0.192^∗^	0.175^∗^	0.200^∗^	0.189^∗^	0.066	0.205^∗^	0.831^∗^	0.872^∗^
	*p*	0.002^∗^	< 0.001^∗^	< 0.001^∗^	< 0.001^∗^	0.001^∗^	< 0.001^∗^	< 0.001^∗^	0.219	< 0.001^∗^	< 0.001^∗^	< 0.001^∗^

*Note: r*: Pearson coefficient.

^∗^Statistically significant at *p* ≤ 0.05.

### 3.1. Mediation Analysis Results

**FIGURE 2:**
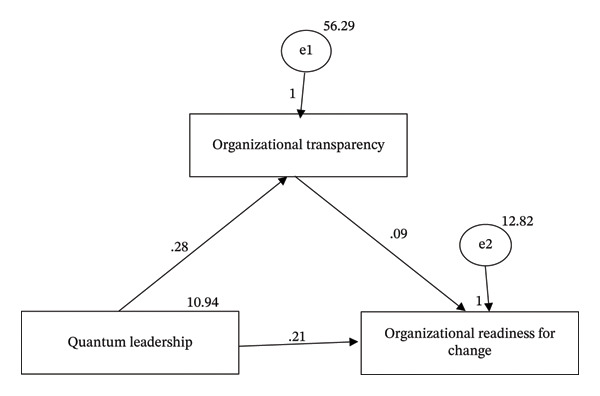
Path analysis of the direct and indirect statistical associations between quantum leadership and organizational readiness for change, with organizational transparency specified as a statistical mediator (*n* = 350).

Table 4 and Figure 2 present the statistical mediation model examining whether organizational transparency statistically mediates the association between quantum leadership and organizational readiness for change. Quantum leadership was positively associated with organizational transparency (path a: *β* = 0.123, *p* = 0.020). Organizational transparency was also positively associated with organizational readiness for change (path b: *β* = 0.182, *p* < 0.001). After organizational transparency was included in the model, quantum leadership remained positively associated with organizational readiness for change (direct association *c* ^′^: *β* = 0.183, *p* < 0.001). The calculated indirect association through organizational transparency was small and positive (*a* × *b* = 0.022), while the total association was 0.205. The model showed acceptable overall fit to the data (CFI = 1.000, IFI = 1.000, RMSEA = 0.078, χ^2^/df = 2.864/3). Although the CFI and IFI values were high, the RMSEA value suggests a reasonable rather than perfect fit. These estimates represent statistical associations and should not be interpreted as causal effects.

**TABLE 4:** Direct, indirect, and total statistical associations between quantum leadership and organizational readiness for change in the model including organizational transparency as a statistical mediator (*n* = 350).

Path	Standardized coefficient (*β*)	SE	CR	*p* value
Path a: Organizational transparency ← Quantum leadership	0.123	0.120	2.325	0.020
Path b: Organizational readiness for change ← Organizational transparency	0.182	0.026	3.514	< 0.001
Direct effect (*c* ^′^): Organizational readiness for change ← Quantum leadership	0.183	0.058	3.533	< 0.001
Indirect effect (*a* × *b*): Quantum leadership ⟶ Organizational transparency ⟶ Organizational readiness for change	0.022	—	—	—
Total effect (*c* ^′^ + *a* × *b*)	0.205	—	—	—

*Note:* CFI = 1.000; IFI = 1.000; RMSEA = 0.078; *χ* ^2^/df = 2.864/3. The indirect effect was calculated as the product of path a and path b: 0.123 × 0.182 = 0.022.

Abbreviations: CFI, comparative fit index; IFI, incremental fit index; RMSEA, root mean square error of approximation.

## 4. Discussion

The present study examined the associations among quantum leadership, organizational transparency, and organizational readiness for change among staff nurses. It also tested whether organizational transparency statistically mediated the association between quantum leadership and readiness for change. Most nurses reported high levels of the three study variables, and the analyses identified small but statistically significant positive associations among them. Given the cross‐sectional, self‐report design, these findings describe patterns of association and do not establish temporal order or causality.

The high level of perceived quantum leadership may be related to nurses′ perceptions that their managers demonstrate adaptive, participatory, supportive, and collaborative leadership attributes. These behaviors are consistent with descriptions of quantum leadership emphasizing flexibility, interconnectedness, creativity, and team‐based problem‐solving in complex healthcare settings. However, because the study relied on nurses′ self‐reported perceptions, the findings do not objectively establish the extent to which these leadership behaviors were practiced. The findings are consistent with previous studies suggesting that quantum leadership attributes may be relevant to nursing and healthcare management [3, 21, 22].

Most nurses also reported a high level of organizational readiness for change. This finding may reflect perceived commitment to organizational change and confidence in the collective ability to implement change initiatives. Because nurses are closely involved in implementing new policies, procedures, technologies, and service improvements, their perceived readiness is an important organizational consideration. Nevertheless, the reported level represents perceptions at a single point in time and should not be interpreted as evidence that future organizational changes would necessarily be implemented successfully. The finding is consistent with previous research suggesting that readiness may be higher when nurses perceive adequate information, preparation, and support [23, 24].

Most nurses also reported a high level of organizational transparency. This finding may indicate that nurses perceived access to relevant information, opportunities for participation, and some degree of accountability in their workplace. Timely, clear, and accurate information may be associated with greater understanding of organizational objectives and lower uncertainty during change. However, the findings do not demonstrate that transparency produced these outcomes, and perceptions of transparency may also be related to other leadership and organizational characteristics. This finding is consistent with studies reporting positive perceptions of transparency and open communication in healthcare and organizational settings [17, 25].

Quantum leadership was positively associated with organizational readiness for change, although the magnitude of the association was small. Nurses who reported higher perceptions of quantum leadership also tended to report greater readiness for change. This pattern may be related to perceived flexibility, communication, participation, and managerial support. However, the cross‐sectional design does not permit the conclusion that quantum leadership increased or produced readiness for change. Reverse, reciprocal, or confounded relationships remain possible. Previous studies have similarly reported associations between leadership and organizational outcomes [26–28].

Organizational transparency was also positively associated with organizational readiness for change. Nurses who reported greater transparency tended to report higher readiness. Access to clear information and opportunities for participation may be related to nurses′ understanding of the rationale, content, and implementation of proposed changes. Transparency may also be associated with trust and lower uncertainty. However, the small correlation suggests that transparency represents only one of several factors that may be related to readiness for change, alongside staffing, workload, resources, training, professional relationships, and previous change experiences [24, 29–31].

The statistical mediation analysis identified a small positive indirect association between quantum leadership and organizational readiness for change through organizational transparency. Specifically, higher perceived quantum leadership was associated with higher perceived organizational transparency, which was subsequently associated with greater reported readiness for change within the specified model. This pattern is consistent with statistical mediation. However, the indirect association was small, and the simultaneous measurement of all variables means that temporal ordering could not be established. Organizational transparency should therefore be described as a statistical mediator or intervening variable in the tested model rather than as a confirmed causal mechanism. Longitudinal and intervention studies are needed to determine whether changes in leadership practices precede changes in transparency and readiness over time [1, 29].

### 4.1. Strengths and Limitations

The study contributes to the body of knowledge on nursing administration in that it focuses on the relationships between quantum leadership, organizational transparency, and organizational readiness for change, an area that has not been adequately studied. To increase the representativeness of the participating units, the use of a census sampling approach within the selected hospital was additionally used.

There are, however, some constraints to take into consideration. First, the cross‐sectional design does not allow any conclusions to be drawn regarding the temporal order or causality. Second, the use of self‐report questionnaires to measure all variables at one time point may have introduced response bias and common method variance. Although procedural measures were applied to reduce these risks and Harman’s single‐factor test did not indicate a dominant single factor, this test cannot exclude common method variance. Therefore, some of the observed associations may partly reflect shared measurement method rather than relationships among the underlying constructs. Third, the study took place in a single hospital, so it is not necessarily applicable to other health care environments. Third, the associations and indirect effect were small and therefore the findings have limited practical implications. Longitudinal, multisite, or intervention designs should be used in future research to explore if leadership behaviors and transparency lead to readiness for change over time.

## 5. Conclusion

The participating nurses reported relatively high levels of quantum leadership, organizational transparency, and organizational readiness for change. Small positive associations were identified among the three variables. Quantum leadership remained positively associated with organizational readiness for change after organizational transparency was included in the model, and a small positive indirect statistical association was observed through organizational transparency. These findings are consistent with a statistical mediation pattern but do not demonstrate temporal ordering or causality. Given the cross‐sectional, self‐report, single‐hospital design, the results should be interpreted as associations among nurses′ perceptions. Organizational transparency may be relevant to the association between perceived leadership and readiness for change, but it should not be regarded as a confirmed causal mechanism. Longitudinal, multisite, and intervention studies are needed to examine these relationships across settings and over time.

### 5.1. Implications for Nursing Management

Nurse managers and nursing leaders should enhance organizational readiness for change by creating clear, organized, and bidirectional communication systems. Managers should communicate with nurses in a timely and understandable manner before and during organizational change initiatives regarding the purpose of the change, expected outcomes, implementation procedures, responsibilities, timelines, and potential impacts on nursing practice. Nurses should be able to raise concerns, seek clarification, and have their concerns documented with a response from management through regular staff briefings, unit meetings, digital communication platforms and formal feedback mechanisms.

Nursing leaders should also ensure that staff nurses are included in committees, planning groups, policy reviews and discussions about changes that impact clinical practice and working conditions. Nurse representation should not be limited to symbolic participation but should also involve opportunities to participate in decision‐making, to identify barriers to implementation, and to suggest practical solutions. Managers should record the process used to consider staff input and explain the reasons for decisions made, especially when recommendations are not implemented.

The implementation of change should be supported by clearly defined managerial responsibilities, transparent reporting processes and regular evaluation of change implementation. Nurse managers should ensure that everyone knows who is responsible for each action, track progress against agreed indicators, recognize issues with implementation, and take corrective action as needed.

Nursing leaders need to evaluate nurses′ readiness for change before launching major organizational initiatives, such as commitment, confidence, information needs, perceived resources, workload concerns, and anticipated resistance. The results of readiness assessments should inform when, how, what, and how much to train and support staff to implement. Leadership development programs should also build nurse managers′ skills in adaptive leadership, participatory decision making, transparent communication, conflict management, and change management. These recommendations should be taken with a grain of salt and should be implemented and assessed carefully using longitudinal and organizational performance indicators, given the small associations found in this cross‐sectional study.

## Author Contributions

Amal Diab Ghanem Atalla contributed to idea, plan, research, initial draft, and further editing and rewriting of the article. Conceptualization, data collection, statistical analysis, data curation, initial drafting, review, and revision were all handled by Ayman Mohamed El‐Ashry and Mohamed Saad Saleh Ali. Data collection, methodology, research, manuscript review, and editing were under the purview of Duaa Amr Hafez, Makiah Mohammed Shebaili, Ebaa Marwan Felemban, Faygah Shibily, and Wafaa Hassan Mostafa.

## Funding

The authors received no specific funding for this work.

## Disclosure

After reading the manuscript, all authors gave their approval.

## Ethics Statement

Every technique used in this investigation fully complied with the relevant guidelines provided in the Declaration of Helsinki (DoH‐Oct2008). The Research Ethics Committee of the Faculty of Nursing at Damanhour University in Egypt formally accepted and permitted the study, which has reference number IRB/DAMANHOUR/98C/3/2024. Additionally, nursing faculty members who willingly consented to participate in the research gave formal informed consent before beginning the study.

## Consent

The authors have nothing to report.

## Conflicts of Interest

The authors declare no conflicts of interest.

## Data Availability

Upon reasonable request, the corresponding author will make the datasets created and analyzed for this study available.
